# Phenotypic divergence and biometric indices of silond catfish, *Silonia silondia* (Hamilton 1822) populations inhabiting the coastal rivers of Bangladesh

**DOI:** 10.1016/j.heliyon.2022.e12484

**Published:** 2022-12-20

**Authors:** Md. Rahamat Ullah, Md. Arifur Rahman, Muhammad A.B. Siddik, Md. Ariful Alam

**Affiliations:** a Department of Fisheries Biology and Genetics, Patuakhali Science and Technology University, Dumki, Patuakhali, 8602, Bangladesh; b Bangladesh Fisheries Research Institute, Riverine Sub-Station, Khepupara, Patuakhali, 8650, Bangladesh

**Keywords:** Biometric indices, Coastal rivers, Phenotypic divergence, Silond catfish

## Abstract

To observe phenotypic differentiation among populations of Silond catfish, *Silonia silondia* (Hamilton 1822), a multilinear approach was used. A sum of 180 samples from three coastal rivers (Meghna, Payra, and Kirtankhola) of the Bangladesh coast were scooped up to comprehend whether distinct populations of *S. silondia* could be separated from one another due to adaptive divergence. The findings of this study reflect the first attempt to determine the stock structure, morphological characters, length-frequency distributions, length-length relationships, length-weight relationships, condition factors, relative weight, and form factor of *S. silondia* in the coastal rivers of Bangladesh. Important discrepancies between the means of the three populations were revealed using univariate and multivariate analysis of variance (*p* < 0.01). In principal component analysis, the first and second principal components described 83.546% and 4.302% of the total variation, respectively. The step-wise discriminant function analysis revealed two variables that separated the populations significantly. Besides, a dendrogram based on Euclidean distances accurately separated the populations. In a one-way analysis of variance study, nineteen out of twenty-one morphometric characters showed significant variation (*p* < 0.01)among three populations. The length parameters based on the length-length relationships of each sample were found to be highly significant (*p* < 0.01). The length-weight relationships exhibited that the *b* value fluctuated from 2.796 (Kirtankhola) to 3.498 (Meghna). The Fulton's condition factor was estimated in the current study for this species with an average value ranging from 1.12 to 1.35. The calculated form factor values of this species were 0.0016, 0.0054, and 0.0110 for Meghna, Payra, and Kirtankhola river, respectively. Therefore, this study will expectantly inform fisheries taxonomists about the species' current stock structure, intraspecific phenotypic divergences, and aid in its management and conservation in similar ecosystems in Bangladesh and around the world.

## 1 Introduction

Stock identification is a precondition for any study related to the fishery, just as population assembly is a fundamental component of conservation ecology (Thorpe et al., 1995). For identifying a stock, several approaches have been suggested, including the detection of self-sustaining mechanisms inside usual inhabitants, conventional tags, parasites as ordinary labels, otolith understanding, and numerous other molecular indicators i.e., mitochondrial DNA, microsatellite DNA (Cadrin et al., 2004; Mir et al., 2013; Sajina et al., 2011). However, in the course of the scientific explanation of fishes, two main techniques including morphometric and meristic are used (Barriga-Sosa et al., 2004; Pinheiro et al., 2005). Over the past fifty years, morphometrics has been used to effectively differentiate a variety of fish populations all over the world (Dwivedi and Dubey, 2013). The morphometric technique is a low-cost method and is used to classify fish stocks, explain stock status, distinguish among fish populations, and connect the ontogeny of a species with functional morphology (Torres et al., 2010). However, the knowledge of morphometric differentiation is required to provide a thorough understanding of a population's biology. Thus, understanding fish morphometric variations aids in the creation of optimal stock management strategies that respond independently to fishing pressure and other ecosystem dynamics (Beger et al., 2004; Turan, 2004). The technique of morphometry method is convenient for research that clearly shows the variances in size and form when performed in combination with multivariate statistics (Baur and Leuenberger, 2011).

Moreover, knowledge of length-length relationships (LLRs) and length-weight relationships (LWRs) are necessary for fish biology, physiology, ecological stock assessment (Hanif et al., 2017; Rahman et al., 2018; Saha et al., 2019), and stock condition because they could be used to parallelism development in various environments (Cherif et al., 2008). LWRs permit life antiquity and morphological comparisons between the diverse type of fish species, or between fish samples from different environments and/or areas (Petrakis and Stergiou, 1995). The weight and length information of fish are very vital parameters in the assessment of the population dynamic, length and age structures, growth and mortality rates, and well-being of the fish (Hossain et al., 2013). LWRs are used to acquire evidence i.e., length frequency distribution from biomass (Rahman et al., 2020a, Rahman et al., 2020b) and fish state (Petrakis and Stergiou, 1995) for assessment of stock and population management of fish (Garcia, 2010) and compare the fish population's growth history from dissimilar areas (Petrakis and Stergiou, 1995).

The silond catfish, *Silonia silondia* (Hamilton 1822), is an amphidromous fish that belongs to the order Siluriformes (Nelson et al., 2016). The fish is widely dispersed in Bangladesh together with India, Pakistan, and Nepal (Talwar and Jhingran, 1991). It is a migratory catfish that lives in most of Bangladesh's coastal waters including the Meghna, Payra, and other rivers as well as India's Brahmaputra, and adults ascend from estuaries into vast rivers during the monsoon season to breed (Chondar, 1999). Since artificial breeding techniques for this and other important species have not yet been established, captured wild stocks met all the demands (Mishra et al., 2009; Rahman et al., 2020a, Rahman et al., 2020b). The primary reasons for its decline in natural stocks are the loss of habitat and overexploitation (Flura et al., 2018). Despite having previously suffered a population decline, the species is now on the rebound. Furthermore, it is low-resilient to fishing pressures which necessitates the conservation of the wild populations (Devi and Boguskaya, 2009). As a result, documentation of this species is needed for the successful conservation, management, and long-term survival of the fishery (FAO, 2019). Due to a lack of proper scientific research, a huge number of fish species in Bangladesh's coastal area remain unidentified (Hanif et al., 2015). There is no information on the stock structure of the *S. silondia* population in Bangladesh's coastal area. As a result, the current study was conducted to determine morphometric differentiations relying on morphometric features as well as the length-frequency distribution, length-length relationships, length-weight relationships, condition factors, relative weight, and form factor for the first time to distinguish the phenotypic variations and biometrics of *S. silondia* collected from various Bangladeshi coastal rivers.

## 2 Materials and methods

### 2.1 Study area

**Figure 1:**
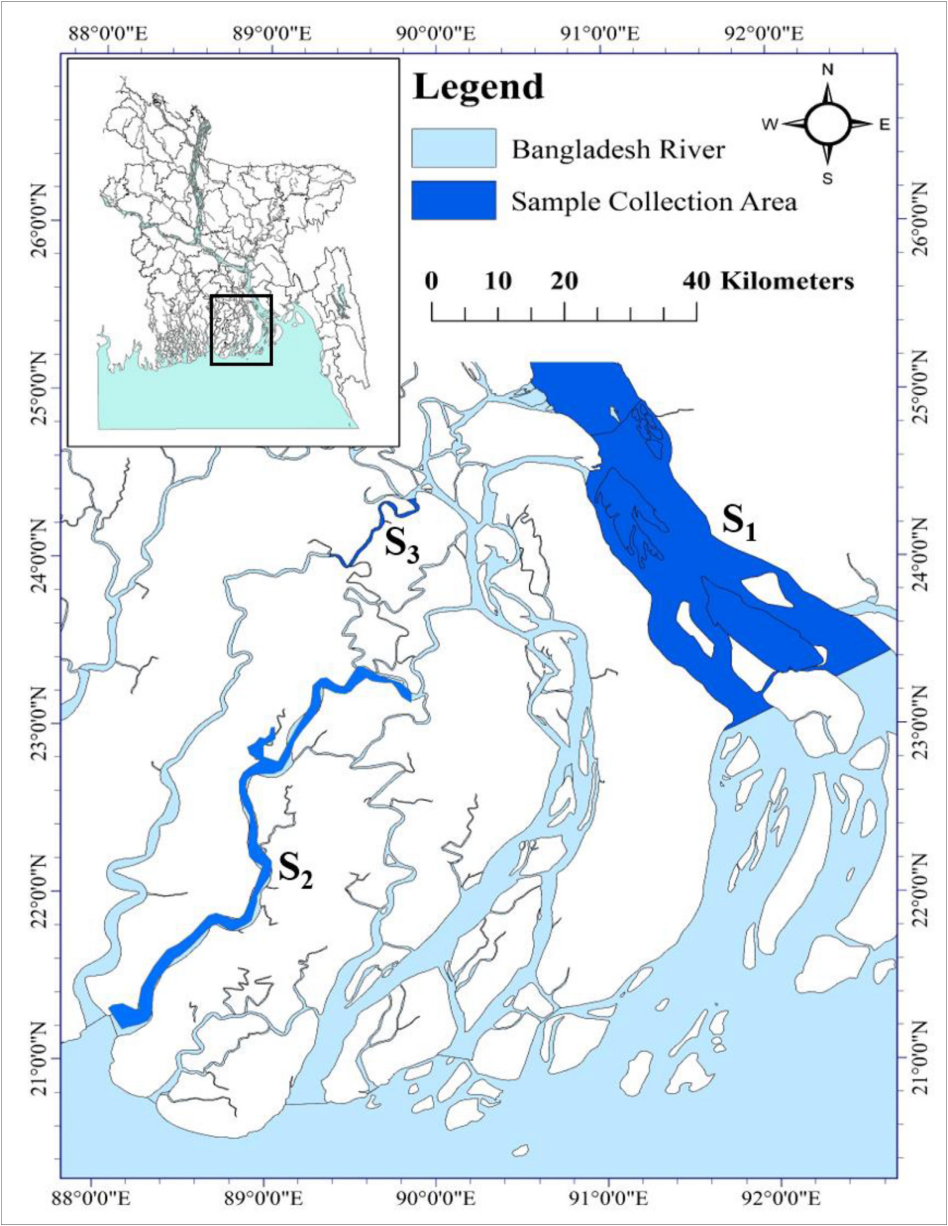
Map depicting the places in the coastal rivers of Bangladesh from where Silond catfish, *Silonia silondia* samples were collected. The deep blue color indicates the location of the sampling site. Sample collection sites were S1, S2, and S3 which indicate the Meghna river in Bhola, the Payra river in Patuakhali, and the Kirtankhola river in Barishal, respectively.

The fish samples were collected from three separate coastal rivers in Bangladesh: the Meghna river in the Bhola district, the Payra river in the Patuakhali district, and the Kirtankhola river in the Barishal district (Figure 1).

### 2.2 Sample collection

Between March 2019 and January 2020, local fishermen helped to collect 180 fish samples. After collecting the sample, intensive morphological studies were done at the Molecular Biology and Conservation Laboratory under the Department of Fisheries Biology and Genetics, Patuakhali Science and Technology University, Bangladesh.

### 2.3 Ethical approval

The experimental protocol and guidelines were maintained according to the Animal Welfare and Ethical Committee of the Faculty of Fisheries at Patuakhali Science and Technology University, Patuakhali, Bangladesh. The research and use of animals for the experiment have been authorized by the Ethical Committee (Ref. No.: PSTU/FoF/EA/2019/04).

### 2.4 Morphometric data collection

**Figure 2:**
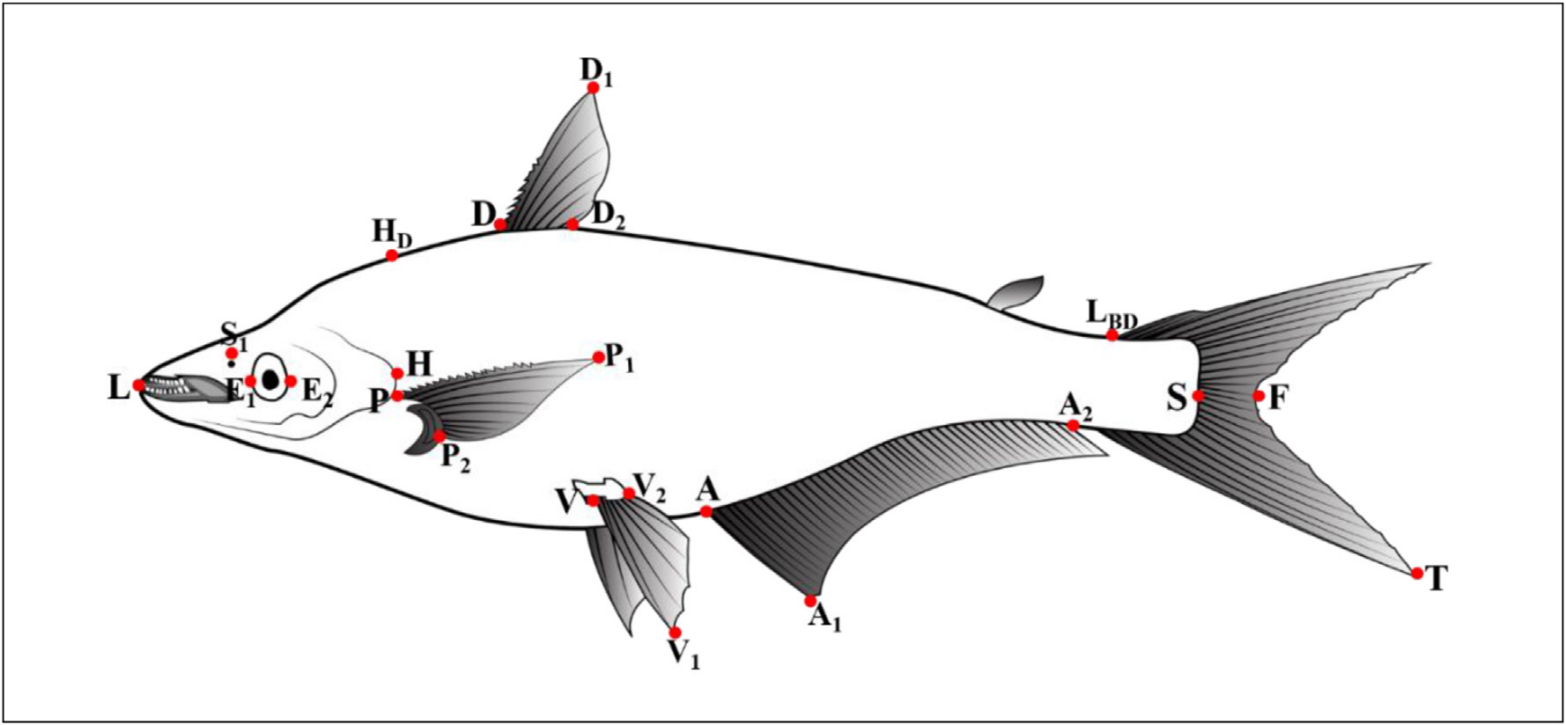
A description of the various morphometric characters examined in Silond catfish, *Silonia silondia* populations inhabiting the coastal rivers of Bangladesh.

By using digital slide calipers a total of 21 morphometric features were measured (Figure 2). The measured characteristics are total length (TL), fork length (FL), standard length (SL), head length (HL), head depth (H_D_↓), highest body depth (D_2_↓), lowest body depth (L_BD_↓), pre-orbital length (LE_1_), post-orbital length (HE_2_), eye diameter (E_1_E_2_), snout length (LS_1_), pre-dorsal length (DL), post-dorsal length (TD_2_), height of dorsal fin (DD_1_), height of pectoral fin (PP_1_), height of ventral fin (VV_1_), height of anal fin (AA_1_), length of dorsal base (DD_2_), length of pectoral base (PP_2_), length of ventral base (VV_2_), length of anal base (AA_2_).

### 2.5 Length-frequency distributions (LFDs)

Length frequency distributions for these three species were created by 1.0 cm intervals of TL. Using the computer software Microsoft Excel, the normal frequency distribution was transformed into the TL frequency distribution.

### 2.6 Length-length relationships (LLRs)

The relationships of length-length with different body lengths were calculated by the least-squares method to suit a basic linear regression model as Y = a + bX, where, Y = various body lengths, X = total length, a = proportionality constant, and b = regression coefficient.

### 2.7 Length-weight relationships (LWRs)

The data was fitting to a possible relationship in the formula, and the length-weight relationships of the fish were calculated (Le Cren, 1951): W = aL^b^, where, W = Weight of fish (g), L = Length of fish, a = Intercept (describe the rate of change of weight with length), *b* = Slope (weight at unit length). The parabolic equation (W = aL^b^) was then transformed into a linear equation using a logarithmic method: Ln W = Ln a + b Ln L.

### 2.8 Conditional indices

The element of the allometric condition factor (K_A_) was calculated by the equation of (Tesch, 1971):

$$K_{A}=W\;/\;L^{b}$$

Where, W = the body weight (g), L = the TL (cm), and *b* = the LWR parameter.

Fulton's condition factor was determined by using the equation of (Froese, 2006; Fulton, 1904):

$$K_{F}=100\times W\;/\;L^{b}$$

where, K_F_ = Fulton's condition factor, W = the weight of the fish (g), L = the total length of the fish in centimeters (cm), and *b* = the value obtained from the length-weight equation.

The relative condition factor (K_R_) was analyzed following the equation of Le Cren (1951):

$$K_{R}=W\;/\;\left(a\times L^{b}\right)$$

where, W = is the body weight (g), L = is the total length (cm), a and *b* = LWR parameters.

The relative weight introduced by Froese (2006) is calculated with the formula:

$$W_{r}=\left(W\;/\;W_{s}\right)\times 100$$

where, W = the weight of a particular individual, W_s_ = the predicted standard weight for the same individual, W_s_ as calculated by W_s_ = aL^b^ (a and *b* values obtained from the composite of length-weight relationships throughout the range of the species).

### 2.9 Form factor (a_3.0_)

The form factor (a_3.0_) of *S. silondia* was estimated through the equation of Froese (2006) as: a_3.0_ = 10^log a – s (b−3)^, where a and b are the regression parameters of LWR, and S is the regression slope of ln a vs. b. The researchers used a mean slope S = -1.358 for calculating the form factor because there was no available information on LWR for this species to estimate the regression (S) of ln a vs. *b*.

### 2.10 Statistical analysis

To normalize the dataset, the size-based morphometric characters were log-transformed. To establish the relationship between standard length and each morphometric character, the log-transformed data were each correlated with fish standard length. After developing the relationship between standard length and morphometric characters, the log-converted characters were adjusted to eradicate the relationship of the variables with distance. This was accomplished by using the allometric technique proposed by Elliott et al. (1995):

$$M_{adj}=M\times {\left(L_{s}/L_{0}\right)}^{\;b}$$

where M is the original measurement, M_adj_ is the size-adjusted measurement, L_0_ is the standard length, L_s_ is the mean of standard length, and parameter b is calculated from experimental data for each character as the slope of log M on log L_0_ using all fish in all populations.

A one-way analysis of variances (ANOVA) for 21 morphometric characters was used to assess the significance of the variations among the three coastal river populations. Principal component (PC) analysis, discriminant function (DF) analysis, and cluster analysis (CA) were used to differentiate the three populations in this study. For population differentiation, principal component (PC) analysis was used to reduce redundancy among morphometric variables and remove redundant autonomous variables (Samaee et al., 2009; Veasey et al., 2001). The difference between and among the entire population was estimated using Wilks' lambda (λ) test. The discriminate function (DF) analysis was used to accurately measure the graded percentage of fish samples. The cross-validation scheme was used to determine the classification functions' credible errors. To investigate the phenotypic relations among populations, a dendrogram based on Euclidean distances was constructed using the Unweighted Pair Group Method (UPGMA) with mathematical average Cluster Analysis (CA) of mathematics means (Sneath and Sokal, 1973). All statistical analyses were performed using SPSS (version 25), PAST (version 4.3), and Excel (Microsoft Office 2019).

## 3 Results

### 3.1 Phenotypic variation

Descriptive statistics are provided in Table 1 for standard length and weight, including mean values for minimum and maximum ranges, as well as standard error, for each population. All the morphometric traits showed significant differences (*p* < 0.01) among the populations of *S. silondia* from coastal rivers of Bangladesh (Table 2) and those significant characters were then used for multivariate analysis (principal component analysis, PCA; discriminate function analysis, DFA; and cluster analysis, CA).

**Table 1:** Descriptive information of Silond catfish, *Silonia silondia* populations inhabiting the coastal rivers of Bangladesh.

Population	Sample no.	Standard length (SL)			Weight (Mean ± SE)
		Min.	Max.	Mean ± SE	
Meghna	50	10.7	13.8	12.20 ± 0.10	20.76 ± 0.61
Payra	50	12.0	17.3	14.88 ± 0.20	46.1 ± 1.85
Kirtankhola	50	7.6	11.3	9.41 ± 0.11	11.48 ± 0.39

Min. = minimum; Max. = maximum; SE = standard error.

**Table 2:** Descriptive information of univariate ANOVA based on morphometric characteristics of Silond catfish, *Silonia silondia* populations inhabiting the coastal rivers of Bangladesh.

Morphometric Characters	Wilks Lambda	F value	p-value
TL	0.201	351.350	*p* < 0.001∗
SL	0.199	356.276	*p* < 0.001∗
FL	0.212	328.462	*p* < 0.001∗
HL	0.270	239.698	*p* < 0.001∗
H_D_↓	0.235	288.402	*p* < 0.001∗
D_2_↓	0.285	222.484	*p* < 0.001∗
L_BD_↓	0.204	344.326	*p* < 0.001∗
LE_1_	0.347	166.262	*p* < 0.001∗
HE_2_	0.278	230.200	*p* < 0.001∗
E_1_E_2_	0.316	191.815	*p* < 0.001∗
LS_1_	0.545	73.774	*p* < 0.001∗
DL	0.179	404.598	*p* < 0.001∗
TD_2_	0.161	459.707	*p* < 0.001∗
DD_1_	0.285	222.232	*p* < 0.001∗
PP_1_	0.261	250.711	*p* < 0.001∗
VV_1_	0.272	236.433	*p* < 0.001∗
AA_1_	0.330	179.983	*p* < 0.001∗
DD_2_	0.888	11.145	*p* < 0.001∗
PP_2_	0.332	177.974	*p* < 0.001∗
VV_2_	0.242	276.913	*p* < 0.001∗
AA_2_	0.217	318.560	*p* < 0.001∗

∗Significant value (*p* < 0.01).

The PCA resulted in the extraction of two principal components. The contribution of a variable to PC was examined for inspecting which morphometric characters generate extreme variance among the populations. In PCA, 21 morphometric dimensions extracted two factors with Eigenvalues >1, elucidating 87.848% of the total variation (Table 3).

**Table 3:** Principal components analysis of Silond catfish, *Silonia silondia* populations inhabiting the coastal rivers of Bangladesh yielded Eigenvalues, percentage of variance, and percentage of cumulative variance.

Component	Principal component 1	Principal component 2
Eigenvalues	17.545	1.103
% of variance	83.546	4.302
Cumulative %	83.546	87.848

**Figure 3:**
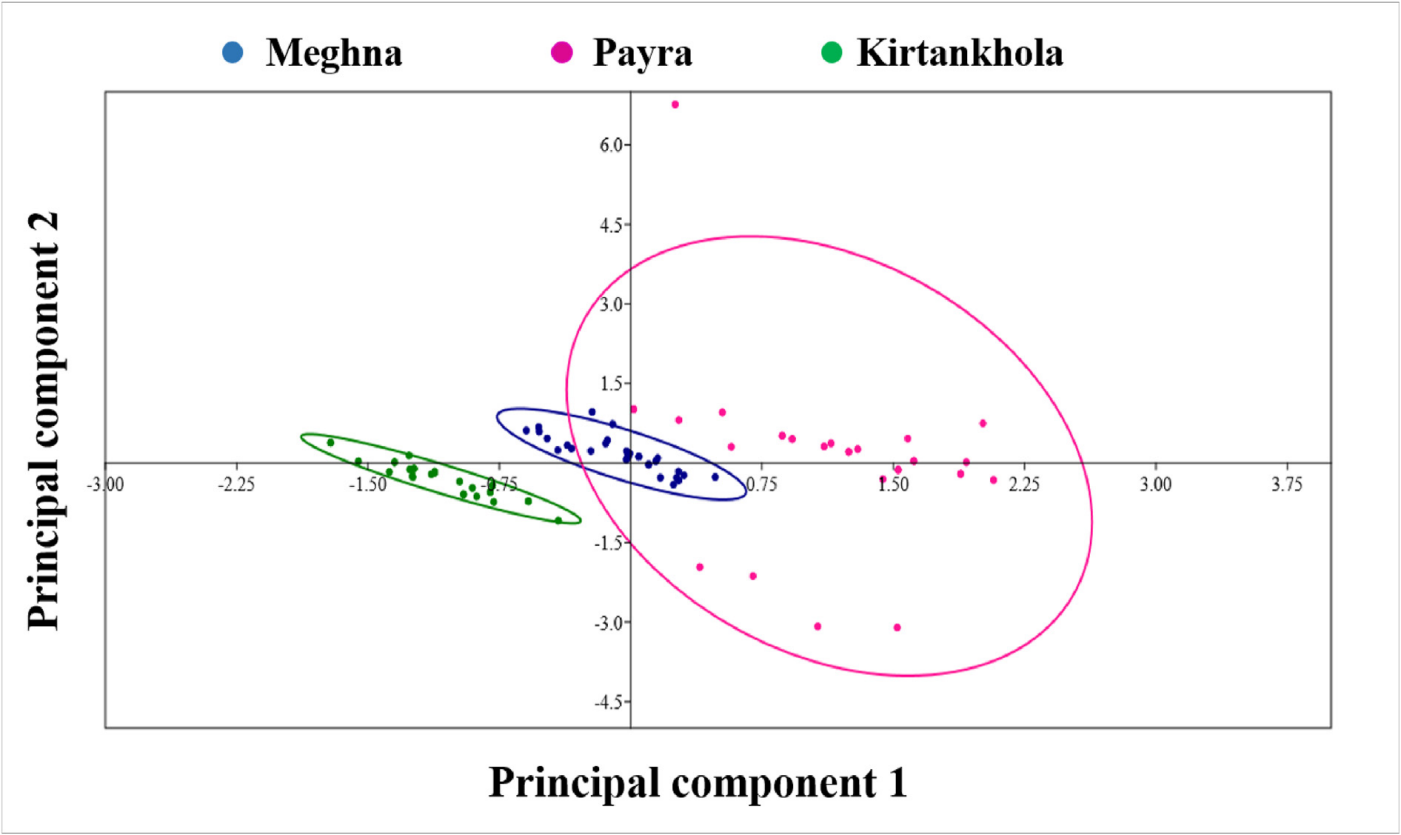
Scatter plot of the scores from PC1 and PC2 for morphometric characters of Silond catfish, *Silonia silondia* populations inhabiting the coastal rivers of Bangladesh.

The first principal component (PC1) defined 83.546% of the total variation and the second principal component (PC2) described 4.302% (Table 4). The most noteworthy loadings on PC1 were TL, SL, FL, HL, H_D_↓, D_2_↓, L_BD_↓, LE_1_, HE_2_, E_1_E_2_, LS_1_, DL, TD_2_, DD_1_, PP_1_, VV_1_, AA_1_, DD_2_, PP_2_, VV_2_, AA_2,_ and on PC2, only DD_2_ was significant. The morphometric traits with an Eigenvalue > 1 were involved and others were omitted in this analysis. Scatter plots of populations concerning the first principal component and the second principal component exposed a visual description of populations (Figure 3) which are grouped into three areas but with high overlap between the Payra and Meghna river but no overlapping with the Kirtankhola river.

**Table 4:** The factor loadings of principal components (PC) based on morphometric characteristics of Silond catfish, *Silonia silondia* populations inhabiting the coastal rivers of Bangladesh.

Characters	PC1 (83.546%)	PC2 (4.302%)
TL	**0.993**	-0.011
SL	**0.891**	-0.147
FL	**0.992**	-0.018
HL	**0.951**	-0.031
H_D_↓	**0.928**	-0.080
D_2_↓	**0.953**	-0.044
L_BD_↓	**0.966**	-0.014
LE_1_	**0.913**	0.039
HE_2_	**0.949**	0.066
E_1_E_2_	**0.867**	-0.120
LS_1_	**0.733**	-0.214
DL	**0.950**	-0.013
TD_2_	**0.938**	-0.003
DD_1_	**0.956**	0.021
PP_1_	**0.971**	-0.002
VV_1_	**0.934**	0.013
AA_1_	**0.914**	0.059
DD_2_	0.413	**0.890**
PP_2_	**0.901**	-0.060
VV_2_	**0.934**	0.073
AA_2_	**0.973**	0.032

Significant loadings >0.5. The highest significant morphometric characters underwritten to the PC1 and PC2 are marked as bold faces.

**Figure 4:**
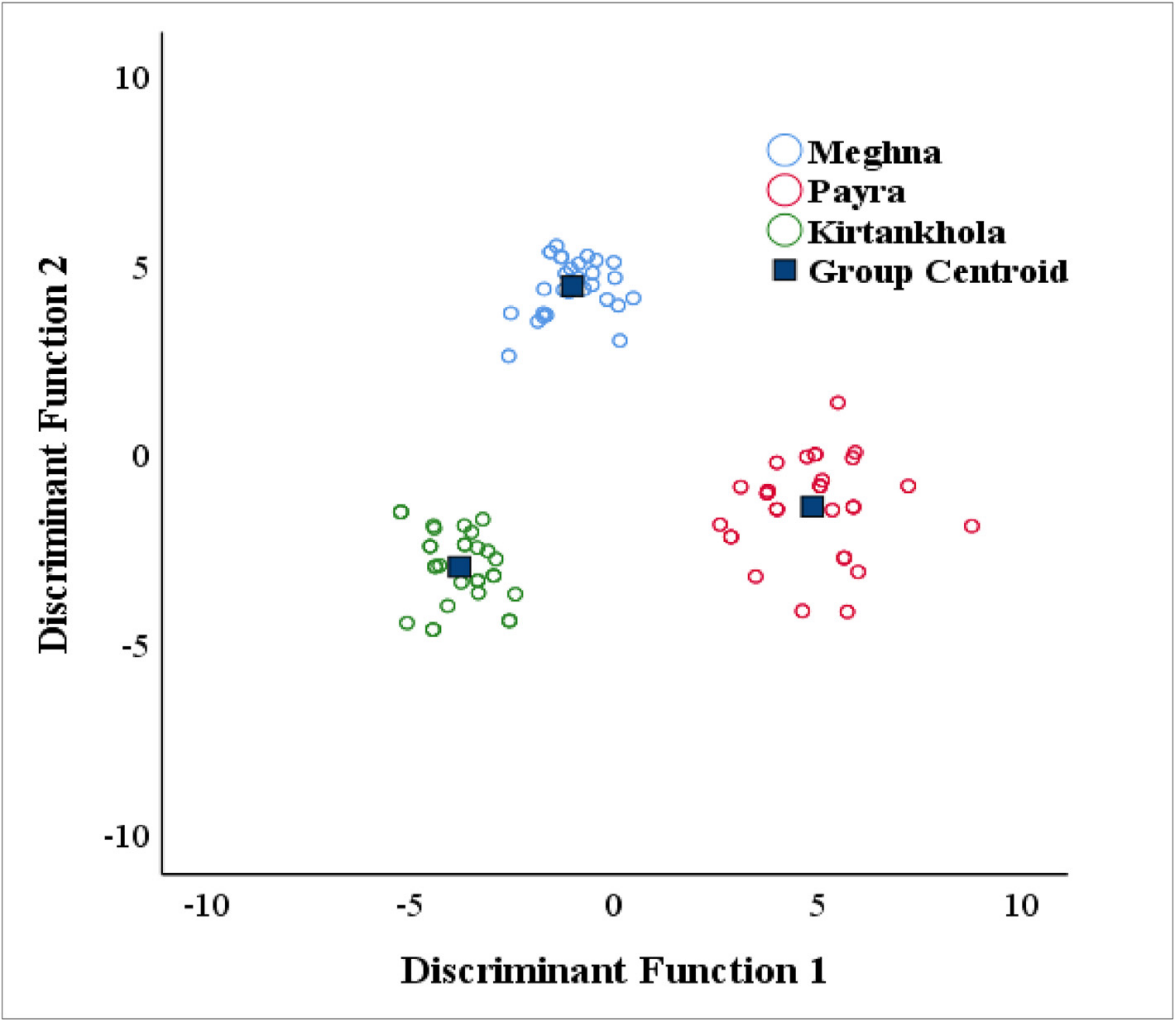
Discriminant function (DF) analysis plot with 21 morphometric variables of Silond catfish, *Silonia silondia* populations inhabiting the coastal rivers of Bangladesh.

Discriminate function analysis (DFA) generated two functions, both with eigenvalues greater than 1 (Table 5). Function 1 had an Eigenvalue of 401.274, accounting for 98.0 percent of the total variance, however, Function 2 had an eigenvalue of 8.273, accounting for just 2.0 percent of the total variance and a combined percentage of 100%. Function 1 was extremely loaded by the characters FL, TL, AA_2_, PP_1_, L_BD_↓, TD_2_, D_2_↓, VV_2_, H_D_↓, VV_1_, SL, AA_1_, PP_2_, and DD_2_. Function 2 was highly loaded by characters DL, HL, E_1_E_2_, HE_2_, DD_1_, LE_1_, and LS_1_. The traits that contributed to Function 1 were highly correlated to the fish's head area, while the traits that contributed to Function 2 were highly correlated to the fish's body and tail regions, but this only accounted for a small amount of group variation as likewise observed in traditional characters. The DF1 versus DF2 plot showed a strong distinction between the three populations and represented 100% of the total variance among them (Figure 4). Wilk's lambda test of discriminant function analysis (DFA) presented a highly significant difference among morphometric traits of all populations (*p* < 0.01) (Table 6).

**Table 5:** Contribution of morphometric measurements to discriminant functions (DF) of Silond catfish, *Silonia silondia* populations inhabiting the coastal rivers of Bangladesh.

Characters	DF1 (98.0%)	DF2 (2.0%)
Eigenvalue	401.274	8.273
% of Variance	98.0	2.0
Canonical Correlation	0.999	0.945
FL	0.788∗	0.085
TL	0.685∗	-0.017
AA_2_	0.205∗	-0.012
PP_1_	0.199∗	-0.196
L_BD_↓	0.182∗	-0.130
TD_2_	0.159∗	0.111
D_2_↓	0.137∗	0.009
VV_2_	0.122∗	-0.076
H_D_↓	0.122∗	-0.101
VV_1_	0.111∗	0.066
SL	0.100∗	0.062
AA_1_	0.097∗	0.019
PP_2_	0.096∗	0.069
DD_2_	0.020∗	-0.016
DL	0.296	-0.527∗
HL	0.187	0.246∗
E_1_E_2_	0.096	0.211∗
HE_2_	0.175	-0.193∗
DD_1_	0.155	-0.169∗
LE_1_	0.102	0.121∗
LS_1_	0.049	0.076∗

∗ Largest absolute correlation between each variable and any discriminant function.

**Table 6:** Results of Wilks’ lambda test for verifying differences among populations of Silond catfish, *Silonia silondia* populations inhabiting the coastal rivers of Bangladesh.

Test of Functions	Wilks' Lambda	Chi-square	Sig.
1 through 2	0.000	1373.448	<0.0001
2	0.108	371.927	<0.0001

The discriminant function analysis revealed that 100% *S. silondia* was originally correctly classified in their respective groups indicating that the populations were correctly classified in their original groups (Table 7). The cross-validation research protocol yielded almost the same results as the PCA results for the populations of three coastal rivers.

**Table 7:** Percentage of specimens classified into each group and after cross-validation for morphometric data of Silond catfish, *Silonia silondia* populations inhabiting the coastal rivers of Bangladesh.

Parameters	River	Meghna	Payra	Kirtankhola	Total
Original	Meghna	100.0	0.0	0.0	100.0
	Payra	0.0	100.0	0.0	100.0
	Kirtankhola	0.0	0.0	100.0	100.0
Cross-validated	Meghna	100.0	0.0	0.0	100.0
	Payra	0.0	100.0	0.0	100.0
	Kirtankhola	0.0	0.0	100.0	100.0

100.0% of original grouped cases are correctly classified. Cross-validation is done only for those cases in the analysis. In cross-validation, each case is classified by the functions derived from all cases other than that case. 100.0% of cross-validated grouped cases were correctly classified.

**Figure 5:**
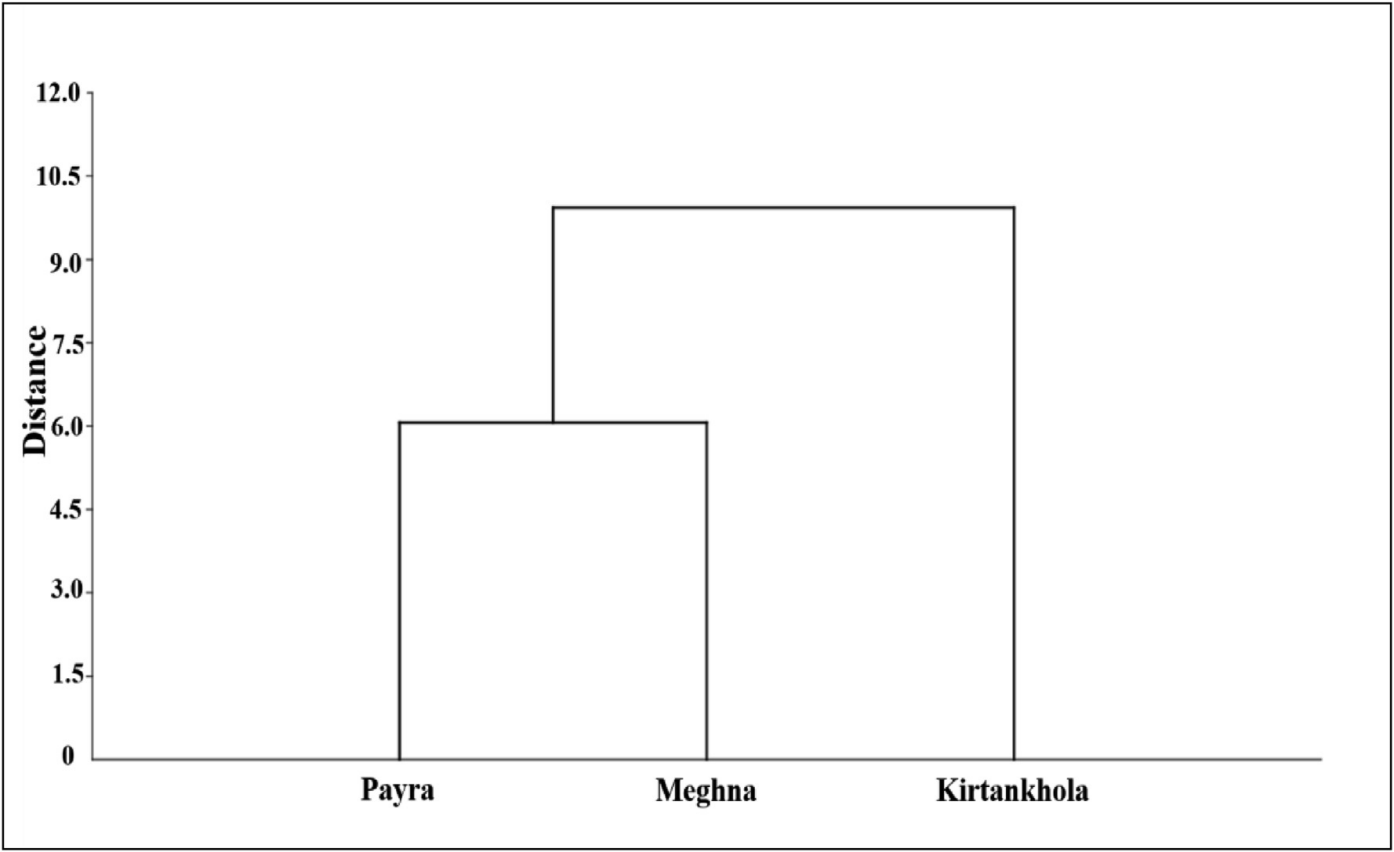
A dendrogram derived from cluster analyses of morphometric measurements based on Euclidean distance for Silond catfish, *Silonia silondia* populations inhabiting the coastal rivers of Bangladesh.

The unweighted pair group method with arithmetic mean (UPGMA) analysis revealed two major clades: the first includes populations from the Meghna and Payra rivers, and the second includes populations from the Kirtankhola river (Figure 5). The Kirtankhola river population was isolated from the others, according to the dendrogram.

### 3.2 Length-frequency distributions (LFDs)

**Figure 6:**
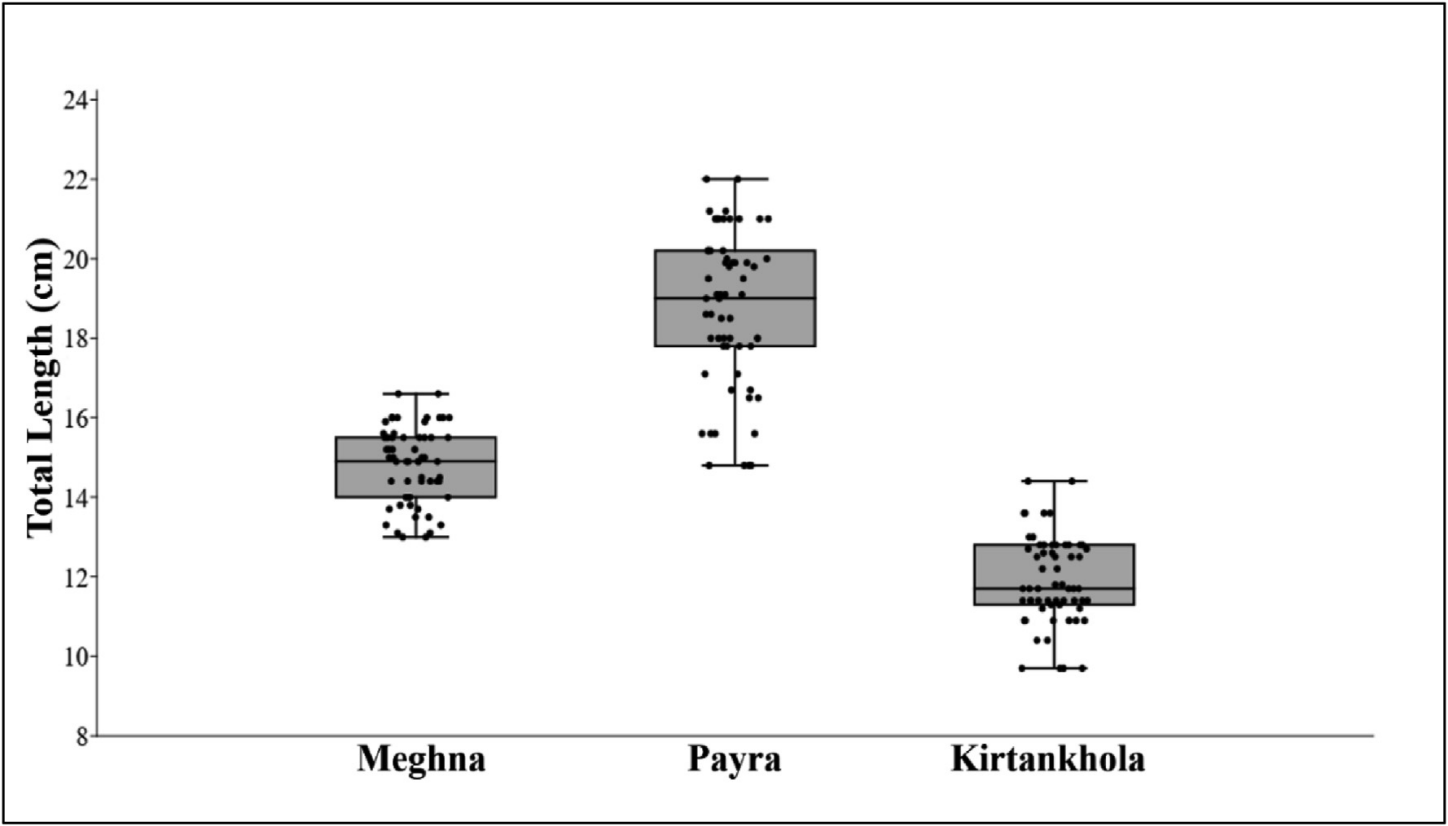
The length-frequency distribution of Silond catfish, *Silonia silondia* inhabiting the coastal rivers of Bangladesh.

The length-frequency distributions (LFDs) of *S. silondia* showed that the minimum and maximum lengths of samples were 13 cm and 16.6 cm TL, 14.8 cm and 22 cm TL, 9.7 cm and 14.4 cm TL with a mean value of 14.84 cm, 18.72 cm, 11.91 cm for Meghna, Payra, and Kirtankhola river, respectively (Figure 6).

### 3.3 Length-length relationships (LLRs)

The LLRs that mean the relations between TL, FL, and SL for *S. silondia* specimens along with regression parameters p and q, and their 95% confidence limit with the coefficient of determination (r^2^) are given in Table 8. All LLRs were extremely significant (*p* < 0.001) with the most coefficients of determination values.

**Table 8:** Descriptive information and estimated parameters of the length-length relationships of Silond catfish, *Silonia silondia* populations inhabiting the coastal rivers of Bangladesh.

Species	Equation	n	a±SE	b±SE	95% CL a	95% CL b	r^2^
Meghna	TL = *p* + *q*×SL	60	0.344 ± 0.31	1.188 ± 0.03	-0.277–0.966	1.137–1.239	0.974
	TL = *p* + *q×*FL		0.439 ± 0.23	1.110 ± 0.02	-0.028–0.905	1.074–1.146	0.985
	FL = *p* + *q×*SL		-0.099 ± 0.16	1.072 ± 0.01	-0.427–0.229	1.045–1.099	0.991
Payra	TL = *p* + *q*×SL	60	0.054 ± 0.34	1.254 ± 0.02	-0.619–0.727	1.209–1.299	0.982
	TL = *p* + *q×*FL		0.720 ± 0.23	1.109 ± 0.01	0.255–1.186	1.080–1.138	0.991
	FL = *p* + *q×*SL		-0.565 ± 0.26	1.129 ± 0.02	-1.076–0.054	1.094–1.163	0.987
Kirtankhola	TL = *p* + *q*×SL	60	-0.022 ± 0.20	1.268 ± 0.02	-0.423–0.380	1.225–1.310	0.984
	TL = *p* + *q×*FL		0.121 ± 0.18	1.139 ± 0.02	-0.231–0.472	1.105–1.173	0.987
	FL = *p* + *q×*SL		-0.043 ± 0.19	1.104 ± 0.02	-0.425–0.338	1.064–1.144	0.981

Note: n, sample size; TL, total length; FL, fork length; SL, standard length; p, intercept; q, slope; SE, standard error; CL, confidence limit; r^2^, coefficient of determination.

### 3.4 Length-weight relationships (LWRs)

A Descriptive statistic and assessed parameters of weight and length including sample sizes (n), regression parameters of a and b of the LWR, and their 95% confidence limit with the coefficient of determination (r^2^) of *S. silondia* are presented in Table 9. All relationships were highly significant (*p* < 0.01) with r^2^ values of the species. In the current study, the calculated growth coefficient of the *b* value of LWRs ranged from 2.759 in the Kirtankhola river to 3.498 in the Meghna river*,* and the coefficients of determination (r^2^) ranged from 0.898 in the Meghna river to 0.979 in the Payra river. The LWRs of *S. silondia* indicated an isometric growth in the Payra river (*b* = 3.00), Negative allometric growth in the Kirtankhola river (*b* < 3.00), and positive allometric growth in the Meghna river (*b* > 3.00).

**Table 9:** Descriptive information and estimated parameters of the length-weight relationships of Silond catfish, *Silonia silondia* populations inhabiting the coastal rivers of Bangladesh.

Species	Equation	n	a ±SE	b ± SE	95% CL a	95% CL b	GP	r^2^
Meghna	BW = a×TL^b^	60	-2.789 ± 0.171	3.498 ± 0.146	-3.131 to -2.448	3.206–3.789	A+	0.909
	BW = a×SL^b^		-2.451 ± 0.166	3.461 ± 0.153	-2.784 to -2.119	3.155–3.767	A+	0.898
	BW = a×FL^b^		-2.498 ± 0.160	3.419 ± 0.144	-2.818 to -2.177	3.131–3.707	A+	0.907
Payra	BW = a×TL^b^	60	-2.271 ± 0.076	3.081 ± 0.060	-2.424 to -2.119	2.961–3.200	I	0.979
	BW = a×SL^b^		-1.983 ± 0.091	3.096 ± 0.077	-2.164 to -1.801	2.941–3.251	I	0.965
	BW = a×FL^b^		-1.937 ± 0.080	2.962 ± 0.066	-2.097 to -1.777	2.830–3.095	A-	0.972
Kirtankhola	BW = a×TL^b^	60	-1.958 ± 0.074	2.796 ± 0.069	-2.107 to -1.809	2.658–2.935	A-	0.966
	BW = a×SL^b^		-1.636 ± 0.090	2.759 ± 0.092	-1.816 to -1.456	2.574–2.944	A-	0.939
	BW = a×FL^b^		-1.789 ± 0.071	2.798 ± 0.070	-1.933 to -1.646	2.657–2.939	A-	0.965

Note: n, sample size; BW, body weight; TL, total length; FL, fork length; SL, standard length; a, intercept; b, slope; SE, standard error; CL, confidence limit; GP, growth pattern; A+, positive allometric; A-, negative allometric; I, isometric; r^2^, coefficient of determination.

### 3.5 Conditional indices

The estimated allometric condition factor (K_A_), Fulton's condition factor (K_F_), relative condition factor (K_R_), and relative weight (W_r_) were shown in Table 10. The highest K_A_ of *S. silondia* was found in the Kirtankhola river followed by the Payra river and Meghna river. In the case of K_F_, the highest K_F_ of *S. silondia* was found in the Payra river followed by Kirtankhola river and Meghna river. On the other hand, in K_R_, the highest K_R_ of *S. silondia* was found in the Kirtankhola river followed by the Payra river and Meghna river. The calculated values of relative weight (W_r_) for *S. silondia* from three coastal rivers populations ranged from 85.59 to 113.93, with a mean value of 99.82 ± 7.37 in Meghna river, 93.95 to 115.05, with a mean value of 99.99 ± 5.09 in Payra river, 89.56 to 107.53, with a mean value 99.63 ± 4.92 in Kirtankhola river which was not different or slightly different from 100 (Table 10).

**Table 10:** Conditional indices of Silond catfish, *Silonia silondia* populations inhabiting the coastal rivers of Bangladesh.

Species	CF	Min	Max	Mean ± SD	95% CL
Meghna	K_A_	0.0014	0.0018	0.0016 ± 0.0001	0.0017–0.0016
	K_F_	0.9776	1.3225	1.1222 ± 0.0942	1.0979–1.1465
	K_R_	0.25	0.28	0.27 ± 0.01	0.266–0.272
	W_r_	85.59	113.93	99.82 ± 7.37	96.751–102.893
Payra	K_A_	0.0050	0.0062	0.0054 ± 0.0003	0.0053–0.0055
	K_F_	1.1969	1.6138	1.3516 ± 0.0882	1.3288–1.3743
	K_R_	0.33	0.36	0.35 ± 0.01	0.342–0.350
	W_r_	93.95	115.05	99.99 ± 5.09	97.875–102.115
Kirtankhola	K_A_	0.0098	0.0118	0.0109 ± 0.0005	0.0107–0.0112
	K_F_	1.2268	1.4961	1.3511 ± 0.0933	1.3270–1.3752
	K_R_	0.42	0.45	0.43 ± 0.01	0.431–0.439
	W_r_	89.56	107.53	99.63 ± 4.92	97.580–101.683

Note: K_A_, allometric condition factor; K_F_, Fulton's condition factor; K_R_, relative condition factor; W_r_, relative weight; Min, minimum; Max, maximum; SD, standard deviation; CL, confidence limit for mean values.

### 3.6 Form factor (a_3.0_)

The calculated form factor (a_3.0_) values of *S. silondia* were 0.0016, 0.0054, and 0.0110 for the Meghna, Payra, and Kirtankhola rivers, respectively which indicated elongated body shape.

## 4 Discussion

The morphological difference is common between and within stocks because of the division of stock within the ecosystems of natural territory. Morphological differences were found to be highly significant among the stocks of the Meghna, Payra, and Kirtankhola rivers in this research. Environment, genetic diversity, human activities, and ecology can all play a role in the major differences. Furthermore, the fish are highly sensitive to changes in their environment (food availability, temperature), and it respond by changing their morphometric characteristics as required (Mir et al., 2013; Wimberger, 1992). Phenotypic plasticity may be attributable to genetic differences between populations, and several studies have been published on morphometric and meristic character measurements on different fish species, revealing differences due to ecology, geography, and human activities (Pinheiro et al., 2005; Robinson and Wilson, 1996). Morphometric differences between stocks are to be expected since they were collected from various locations and may have descended from different ancestors, as well as their adaptability (Hossain et al., 2010). However, it is often tough enough to understand the reasons for morphological variations among stocks (Poulet et al., 2004).

Multivariate analysis of morphometric characters, on the other hand, is a simple and efficient procedure for determining the discrimination of many fish (Overton et al., 1997). In addition, to authenticate our primary analysis, this study has done the multivariate analysis, including PCA, DFA, and CA statistical analysis. In PCA, a factor loading higher than 0.30 is regarded as significant, higher than 0.40 is regarded as more significant, and factor loadings of 0.50 or higher are regarded as very significant (Lombarte et al., 2012). Significant factors in the current study were described as those with loadings greater than 0.50.

The populations were distinguished by multivariate analysis PCA, which revealed that populations had a high degree of relation between the Payra and the Meghna rivers but no overlap with the Kirtankhola river in terms of morphometric traits. Fish populations from the Payra and Meghna rivers were morphometrically close, which may be attributed to migration and mixing between the two populations. As the Meghna and Payra river are connected with the Bay of Bengal and Kirtankhola aren't connected with the Bay of Bengal, the stock of the Kirtankhola river was dissimilar from the other stocks. Furthermore, the Kirtankhola river population is geographically separate from the others, while the other two rivers are not too geographically isolated. The distance between the rivers, the different geographical locations, and the environmental constraints that each population faces may all contribute to this inter-population disparity. Díaz de Astarloa et al. (2011) used multivariate analysis to explain the morphological variations between two species of hake in Argentinean waters. Stocks morphometric features might be different following position, environment, and ecological fluctuations, according to previous studies (Cabral et al., 2003; Díaz de Astarloa et al., 2011; Hermida et al., 2005; Swain and Foote, 1999).

Discriminant function analysis (DFA) may be a good way to distinguish different populations of similar species, which could be troublesome for stock managing systems (Karakousis et al., 1991). In our current research, DFA was used to correctly identify a large number of individuals into their categories, and PCA was used to validate this distinction. The ability of morphometric traits to reveal high flexibility in response to fluctuations in ecological circumstances is well known (Swain and Foote, 1999). During an analysis of morphometric dissimilarity in vandace (*Coregonus albula*) in the Lake of Lithuania, similar findings were observed (Kaupinis and Bukelskis, 2010). Habitat variations had a big impact on morphological discrimination in different populations (Ferrito et al., 2007).

During discriminant analysis, all of the initial clustered cases were correctly categorized 100 percent of the time. Turan et al. (2005) correctly identified 78 percent of six *Clarias gariepinus* stocks in a related experiment. Asaduzzaman et al. (2021) likely observed a high degree of accuracy in classifying the specimens into their original populations, with cross-validated classes of 100% and 97% for *S. olivacea* populations and 99% and 95% for *S. serrata* populations.

In this research, clustering investigation based on Euclidean distances using a UPGMA cluster separated the Kirtankhola river population from the Meghna and Payra river populations, which could be linked to geographic detachment as geographic separation causes morphological variations (Yamamoto et al., 2006). The Meghna and Payra rivers are directly linked to the Bay of Bengal, while the Kirtankhola river is not. When a species' distribution is more or less constant over a range, the equilibrium between gene flow and the forces that trigger stock differentiation, i.e., genetic drift or differential selection, can result in inclines, in which genetic differentiation increases as geographic distance increases (Pinheiro et al., 2005).

The size effect for morphometric information was successfully extracted using allometric transformation in the current study, indicating that morphological variations of *S. silondia* within the coastal rivers of Bangladesh could be related to head and body shape differences. As a result, differences in geographical location, and food abundance along abiotic characteristics such as salinity, tidal fluctuation, dissolved oxygen, etc. may be responsible for the separation of the three populations of *S. silondia*.

The length-frequency distributions depict the population structure of fish, which offers valuable knowledge for fisheries management. Understanding the size structure of fish populations is critical for understanding development, reproduction, and recruitment, with changes in size acting as an early indicator of disturbance. This study's length-frequency distributions provide snapshots of the size structure of the species found in Bangladesh's coastal rivers. The minimum size (TL) of *S. silondia* was 13 cm, 14.8 cm, and 9.7 cm in Meghna, Payra, and Kirtankhola river, respectively in total length and the maximum size was 16.6 cm, 22 cm, and 14.4 cm in Meghna, Payra and Kirtankhola river, respectively in total length that is smaller than the reported maximum value of 26 cm (Bhuiyan, 1964), and 80 cm (Rahman, 2005). The non-appearance of larger individuals in the sampling areas in this study may be attributed to overfishing in Bangladesh's coastal waters. Furthermore, unlike previous studies (Rao and Dwivedi, 1989; Hossain, 2010) in which different fishing techniques induced biased estimation of various population parameters, including the maximum size, local fishermen used specific fishing gear with similar mesh sizes, resulting in similar catch composition (Hossain et al., 2012).

The regression equation of length-length relationships is established for *S. silondia* to assess the symmetrical growth concerning body length i.e., TL vs SL, TL vs FL, and FL vs SL relationships. In the current study, the length-length relationships were highly significant, with all of the coefficient of determination (r^2^) values being >0.974. Results comparisons were not possible due to an absence of available works on the length-length relationships for *S. silondia*. Differences in length-length relationships can be due to variances in ecological conditions of animal habits, variances in animal physiology, or both (Hossain et al., 2013).

The parameter *b* (slope) defines the allometric or isometric growth rate, which is defined by genetic factors, and if it remains stable and assumes values equivalent to or equal to 3.0, it means the individual's shape and ontogenetic growth remain unchanged. In the present study, the parameter *b* (slope) values for *S. silondia* in the Meghna river (*b* = 3.498), in the Payra river (*b* = 3.081), in the Kirtankhola river (*b* = 2.796) were within the expected range between 2.5 and 3.5 (Froese, 2006; Garcia, 2010; Islam et al., 2017; Rahman et al., 2012). Usually close to 3.0 and constant at '3′ in an ideal fish (Carlander, 1969), and also in the majority of the cases, the value was not equal to 3 (Hile, 1936) but also, they can vary in acceptable ranges between 2.0 and 4.0 (Hile, 1936; Hossain et al., 2012; Tesch, 1971). In the current study, *S. silondia* of the Payra river showed isometric growth while populations of the Meghna river showed positive allometric growth, and populations of Kirtankhola river showed negative allometric growth. By observing previous and present studies the variation in the value of exponent '*b*' or regression coefficient which changes may be attributed to differences in sampling, season, sample size, or length ranges. It can be by following factors influence most areas, locality or habitat, sex (Hossain, 2010; Hossain et al., 2012; Hossen et al., 2017), temperature, feeding rate, overfishing, food competition (Rahman et al., 2012; Tesch, 1971), behavior (active or passive swimmer), ecological factors (availability of forage organisms) (Le Cren, 1951), preservation techniques, differences in the observed length ranges of the captured specimens (Hossain et al., 2012), water flow, maturity, stage of maturity, metamorphosis, season or seasonal effect, gonadal development, growth phase and trophic potential of rivers and ponds of animals (Alam et al., 2012; Garcia, 2010).

Conditional indices (K_A_; K_F_; K_R_) were estimated where the values of a fish mainly consider current bio-physical (physical and biological) circumstances and become agitated by interaction among feeding conditions, pathogenic infections, and physiological factors (Froese, 2006; Hossain et al., 2012; Le Cren, 1951) and also food reserves as an indicator of the general fish condition. K_F_ value of species was >1 showing their perfect condition whereas, its value < 1 reflects that the well-being of the fish is not in a good condition (Froese, 2006; Hile, 1936; Hossain et al., 2012; Islam et al., 2017; Tesch, 1971). So, K_F_ is the best condition index for assessing well-being among other condition factors which were significantly correlated with TL and BW. The condition factor (K_F_) was observed in the present study for *S. silondia* with an average value of 1.1222, 1.3516, and 1.3511 in Meghna, Payra, and Kirtankhola river, respectively. The results indicate that fish species are doing well in the coastal rivers of Bangladesh. Relative weight (W_r_) is a good predictor of prey availability (Porath and Peters, 1997). If a fish is heavier than its relative weight, it has more energy reserves available for normal activities, development, and reproduction (Murphy et al., 1991). A relative weight of less than 80 is considered very slim, 90 is considered normal, and greater than 100 is considered obese (Blackwell et al., 2000; Hossain et al., 2013). The W_r_ values for an individual or population when >100 suggest such as low prey availability or high predatory density; whereas values < 100 indicate a prey surplus or low predatory density (Hossain et al., 2013). In the current study, the W_r_ values for *S. silondia* were near 100 which results indicated the species are heavier and fat with good health conditions and reproduction. These results were confirmed by (Hossain et al., 2013; Murphy et al., 1991; Rahman et al., 2012) where were reported various species. However, the current study was to carry out a comprehensive description of W_r_ for the *S. silondia* in the coastal rivers of Bangladesh.

In Bangladeshi waters, there is only a limited study on fish form factor (Hossain et al., 2012; Rahman et al., 2012). The form factor (a_3.0_) can be used to see whether a population's or species' body shape is substantially different from others (Froese, 2006). *S. silondia* form factors were 0.0016, 0.0054, and 0.0110 for the Meghna, Payra, and Kirtankhola rivers, respectively, suggesting an elongated body shape typical of many riverine fishes. Nevertheless, since there is no information in the literature about the form factor of this species, this is the first report on *S. silondia*, which will be useful for future research.

## 5 Conclusion

Significant morphometric variations, discrete conditional indices, and form factors among three distinct populations of *S. silondia* were evaluated in the current analysis. On its alone, morphometric data cannot encompass any of the solutions. Aside from genetic influences, morphometric differences among *S. silondia* populations may be affected by several external factors such as geographical position, diet, and other environmental factors. As a result, more research is needed to understand the impact of external influences on this species' morphometric separation, as well as gonadal development to sustain and conserve this species.

## Declarations

### Author contribution statement

Md. Rahamat Ullah: Performed the experiments; Analyzed and interpreted the data; Wrote the paper.

Md. Arifur Rahman: Conceived and designed the experiments; Analyzed and interpreted the data; Wrote the paper.

Muhammad A. B. Siddik: Analyzed and interpreted the data; Wrote the paper.

Md. Ariful Alam: Conceived and designed the experiments; Analyzed and interpreted the data; Contributed reagents, materials, analysis tools or data.

### Funding statement

Md. Arifur Rahman was supported by Research and Training Center (RTC) under 10.13039/501100014587Patuakhali Science and Technology University and University Grants Commission of Bangladesh [5921, Fish-01].

### Data availability statement

Data included in article/supp. material/referenced in article.

### Declaration of interests statement

The authors have no competing interest to declare.

### Additional information

No additional information is available for this paper.
